# Shedding LIGHT (TNFSF14) on the tumor microenvironment of colorectal cancer liver metastases

**DOI:** 10.1186/1479-5876-11-70

**Published:** 2013-03-20

**Authors:** Jian Zhong Qin, Vivek Upadhyay, Bellur Prabhakar, Ajay V Maker

**Affiliations:** 1 Department of Surgery, University of Illinois at Chicago, Division of Surgical Oncology, 835 S. Wolcott MC790, Chicago, IL 60612, USA; 2 Department of Microbiology and Immunology, University of Illinois at Chicago, 835 S. Wolcott, Chicago, IL 60612, USA; 3 School of Life Science and Biotechnology, Dalian University, Dalian, China

**Keywords:** Colorectal liver metastases, Immunotherapy, LIGHT, Tumor infiltrating lymphocytes

## Abstract

**Background:**

T-cell infiltration in primary colon tumors is associated with improved patient survival. Preliminary data supports a similar association in colorectal liver metastases (CRLM), and we previously identified increased CRLM expression of the immunostimulatory cytokine LIGHT (TNFSF14) to be related to improved patient prognosis. Therefore, mechanisms to augment the T-cell response in CRLM may be a promising treatment modality, however, the tumor immune microenvironment and LIGHT expression in CRLM remains to be characterized.

**Methods:**

Utilizing a syngeneic and immunocompetent model of CRLM, the immune microenvironment was characterized for lymphocyte phenotype, function, and location utilizing flow cytometry, immunoassays, and immunofluorescence microscopy.

**Results:**

CD3+ and CD4+ lymphocytes were decreased, and CD8+ cells were increased in CRLM compared to control liver. When present, greater populations of tumor infiltrating lymphocytes (TIL) were found peritumoral than intratumoral. The TIL expressed significantly higher levels of CD69 and CD107a, but lower levels of LIGHT. Cytokine expression profiles revealed increased levels of the T-helper 1 (Th1) cytokines IFN gamma, IL-12, IL-1b, and IL-8 in CRLM compared to control liver tissue. There was no difference in T-helper 2 (Th2) cytokines between the groups.

**Conclusions:**

Characterization of the tumor microenvironment of CRLM revealed that although a limited number of activated T-cells infiltrate the tumor and initiate an immune response, the number of LIGHT + T cells infiltrating the tumor were very low. Techniques to decrease suppressive influences or augment the cytotoxic T-cell response are needed and may be possible through mechanisms that can increase intratumoral TIL LIGHT expression.

## Introduction

Almost 5% of the global population [1] is diagnosed with colon cancer and roughly 608,000 people die from colorectal cancer annually [2]. These numbers are increasing as colorectal cancer incidence rises, possibly secondary to changes in lifestyle patterns [3]. Within the United States, colorectal cancer is expected to be responsible for over 140,000 new cancer cases among men and women, accounting for 8% of cancer deaths in men and 9% of cancer deaths in women [4]. Even though colorectal cancer mortality decreased in the United States between 1976-2000, most of this improvement was secondary to improved screening (53%) while improvements in treatment only decreased mortality by 12% [1]. Therefore, in addition to raising awareness of risk factors and continuing to improve surveillance, there is a need to also improve colorectal cancer therapies.

The majority of patients with advanced colorectal cancer will die of metastatic disease, most commonly to the liver. Colorectal cancer liver metastases (CRLM) develop in 20% of patients with Stage II colon cancer, and 50% of patients with stage III colon cancer [5]. In highly selected cases, surgical resection may provide long-term survival, however, only 10-20% of patients are candidates for surgery, and of those patients who do undergo surgery, only 40% reach 5-year survival [5].

Standard management of diffuse CRLM involves multidrug chemotherapy regimens [6]. In addition to treatment-related toxicity, the chemotherapeutic regimens are limited in that they are often tumoristatic. The development of novel tumoricidal cancer therapies is needed.

The presence of T-cell infiltrates in primary colon tumors is more prognostic than traditional TNM staging and more directly correlates with overall survival [7]. Furthermore, we and others have shown that increased CD4 and CD8 T-cell infiltrates, and decreased Foxp3+ T regulatory cell ratios, in CRLM are also associated with improved overall survival [8,9]. We have also demonstrated in preliminary gene array studies that T cell proliferation is the most significant biological process associated with survival in patients with colorectal cancer liver metastases [10]. Therefore, mechanisms to improve the immunogenicity of CRLM or augment the T-cell response may be a promising treatment modality for these patients.

LIGHT (TNFSF14) is an immunostimulatory cytokine that has been shown to augment the anti-tumor immune response and whose overexpression we identified as being associated with improved overall and recurrence free survival in patients with CRLM [10]. A member of the Tumor Necrosis Factor Superfamily (TNFSF), it bears close homology with lymphotoxin a-β, lymphotoxin α, and Fas ligand. LIGHT is expressed predominantly on lymphocytes, and it principally interacts with two receptors – Lymphotoxin β receptor, located on other lymphocytes, and herpesvirus-entry-mediator (HVEM), which is located mainly on stromal cells [11]. Forced LIGHT overexpression in tumors leads to increased levels of cytotoxic T lymphocytes (CTLs) in and around the tumor and can induce tumor regression [12]. LIGHT may allow CTL’s to overcome the antigenic barrier formed by host cell stroma around the tumor and to mount an anti-tumor response [13]. In order to determine the utility of LIGHT and other immunostimulatory cytokines for CRLM immunotherapy, the tumor microenvironment of CRLM first requires characterization, which has not yet been performed. The purpose of our research is to accurately determine the intra and peri-tumoral immunologic milieu of CRLM and the expression and location of LIGHT within it.

## Methods

### Cell Culture, Cell Staining, and FACS analysis

CT26 murine colorectal carcinoma cell line was obtained from the American Type Culture Collection (ATCC, Manassas, VA, USA) and was grown in RPMI 1640 culture medium (Sigma-Aldrich, St. Louis, MO, USA) supplemented with 10% fetal bovine serum (Invitrogen, Grand Island, NY, USA) and 1% Anti-Anti (Invitrogen, Grand Island, NY, USA). Antibodies for staining leukocytes and FACS analysis included anti-mouse CD3, CD4, CD8, CD25, CD107a, CD69, and Foxp3 (eBioscience Inc., San Diego, CA, USA). Anti-mouse-Light-A647 was internally validated (eBioscience Inc., San Diego, CA, USA). Gating references for lymphocytes were established by forward and side scatter profiles of corresponding peripheral blood mononuclear cells.

Intrahepatic lymphocytes and tumor infiltrating lymphocytes were treated with PMA and Ionomycin and stained for cell surface makers. After fixation with Cytofix/Cytoperm buffer (Becton Dickinson) cells were stained with anti-Light antibody (eBioscience Inc., San Diego, CA, USA). Stained cells were analyzed on a CyAn ADP analyzer (Beckman Coulter, Brea, CA, USA). Events were collected and analyzed using Flow Jo software (Tree Star Incorporated, Ashland, OR, USA).

### Lentivirus production and infection

A bicistronic lentiviral vector (pHIV1SDm-CMV-GFP-P2A-luc) simultaneously expressing GFP and firefly luciferase was utilized. 293FT cells were transfected with the lentiviral vector and pcDNA3-Tat, pHCMV-Rev, pHCMVgagpol, and pHCMV-G (kindly provided by Dr. Jeff Holst, University of Sydney, Sydney, Australia). Using a calcium precipitation method [14], a stably transduced CT26 cell line that expressed both GFP and luciferase was created.

### Mouse model of colorectal cancer liver metastases

Female BALB/c mice weighing 18–20 g were purchased from Charles River Laboratories (Wilmington, MA, USA) and maintained in specific pathogen-free conditions. All animal care and surgical procedures were performed in accordance with protocols approved by the Office of Animal Care and Institutional Biosafety (OACIB) of University of Illinois at Chicago (Chicago, Illinois, USA). Mice were anesthetized and a laparotomy was performed. 1x10^6^ CT26 cells in 0.1ml of PBS or PBS alone (for control group) was injected into the spleen parenchyma followed by splenectomy in both groups. Tumor growth was monitored by bioluminescence imaging of live animals with the Xenogen in vitro imaging system (Caliper Life Sciences, Inc., Hopkinton, MA).

### Isolation of intrahepatic lymphocytes and tumor infiltrating lymphocytes

Mice were euthanized by CO_2_ inhalation. Livers were immediately perfused through the portal vein with 5 mL of PBS and livers were then surgically resected. The metastatic liver tumors (CRLM) were surgically macrodissected at the border between obvious tumor tissue and healthy appearing parenchyma, minced, and incubated in 10 mL RPMI containing 5% FBS, collagenase IV (1 mg/ml, Sigma), and DNase I (50 ug/ml, Sigma); and strained to obtain a single cell suspension. A mononuclear cell-enriched fraction was isolated using Percoll centrifugation media (GE Healthcare Biosciences, Pittsburgh, PA, USA), and cell viability determined by trypan blue exclusion, which was >90%.

### Peripheral blood mononuclear cell (PMBC) isolation

Following euthanasia, mouse peripheral blood was obtained via retroorbital bleed. Blood specimens from 3-4 mice were pooled and loaded onto the top of a Ficoll-Hypaque centrifugation column per manufacturer’s instructions. The lymphocyte-enriched layer was collected and red blood cells were lysed with ACK lysing buffer (Invitrogen, Grand Island, NY, USA).

### Protein extraction from control liver or liver metastases

Fresh surgically dissected control liver or CRLM were immediately frozen in liquid nitrogen and stored in -70°C for protein preparation. Frozen specimens were pulverized (Biopulverizer, BioSpec Products, Bartlesville, OK, USA) and lysed with 1% Triton-X100, 0.5% NP-40, 0.25% Na-deoxycholate, 1 mM EDTA, 1 mM EGTA, 5 mM NaF, 1 mM orthovanadate, 1 uM microcytin, 1 mM AEBSF and complete protease inhibitor cocktail per manufacturer’s instructions (Roche, Indianapolis, IN, USA). Protein concentrations were measured using Bradford reagents (Bio-Rad Laboratories, Hercules, CA, USA).

### Cytokine assay

Cytokine quantification in control liver and CRLM was performed with a mouse Th1/Th2 ultrasensitive immunoassay (Meso Scale Discovery, Gaithersburg, MD, USA) following the manufacturer’s protocol. The kit was used to detect mouse IFN-gamma, IL-1 beta, IL-2, IL-4, IL-5, IL-8, IL-10, and total IL-12 in a sandwich immunoassay format. The plate was read on the SECTOR imager and analyzed with MSD Discovery Workbench Software (Meso Scale Discovery, Gaithersburg, MD, USA).

### Immunofluorescence

Surgically resected control liver and tumors were embedded in OCT (Tissue-Tek, Sakura Finetek, Torrance, CA, USA). For concurrent staining of LIGHT, CD3, CD4, and CD8, 6μm cryostat sections were prepared. Slides were fixed in acetone, blocked in 5% BSA, and incubated with LIGHT affinity-purified goat anti-LIGHT antibody (AF1794, R&D Systems, Minneapolis, MN, USA) followed by affinity purified donkey anti-goat IgG conjugated to FITC (sc-2024 Santa Cruz Biotechnology, Santa Cruz, CA). T-cell markers were stained with Anti-Mouse CD3, CD4, or CD8 conjugated to AlexaFluor® 700 (eBioscience, San Diego, CA, USA). Following secondary antibody incubation, sections were washed and mounted using UltraCruz Mounting Medium containing DAPI (sc-24941, Santa Cruz Biotechnology, Santa Cruz, CA, USA). Ag104 cells that stably overexpressed LIGHT [15] were used as a positive control (gift of Dr. Yang Xin Fu). Ag104 cell monolayers were grown in Lab-Tek Chamber Slides (Nalge Nunc International, Rochester, NY, USA) to 70-80% confluence and then washed, fixed and appropriately stained. As a negative control, CT26 colon carcinoma cells were grown in culture and handled using the same protocol. They were confirmed to show no LIGHT signal via immunofluorescence and verified to express no LIGHT via western blot and FACS analysis (data not shown). Immunofluorescence and photographs were obtained with an Olympus BX51 fluorescence microscope in the RRC Confocal Microscopy Facility (University of Illinois Chicago, Chicago, Illinois, USA). Images were acquired using an Olympus DP71 CCD camera and DP Controller Software (Olympus, Center Valley, PA, USA).

TIL quantification was performed according to our standard clinical pathology protocol for primary colon cancers and CRLM, as has previously been reported and is utilized by many groups [16-18]. In brief, only cells infiltrating between tumor cells were counted and apoptotic cells were not included. The tumor was reviewed at low power and an area with the most TIL or lymphocytes was identified. In this location, five consecutive 400× fields were counted and the mean TIL/high power field for each tumor was then calculated. Peritumoral lymphocytes were considered the lymphoid cuff at the leading edge of the tumor [16].

### Statistical analysis

Data were presented along with the standard error of the mean (SEM) where appropriate. Differences between groups were calculated using the student two–tailed t-test (Microsoft Excel, Redmond, Washington: Microsoft, 2007). Significance was considered for p-values < 0.05.

## Results

### Phenotype and function of T cell subsets in colorectal liver metastases

**Figure 1:**
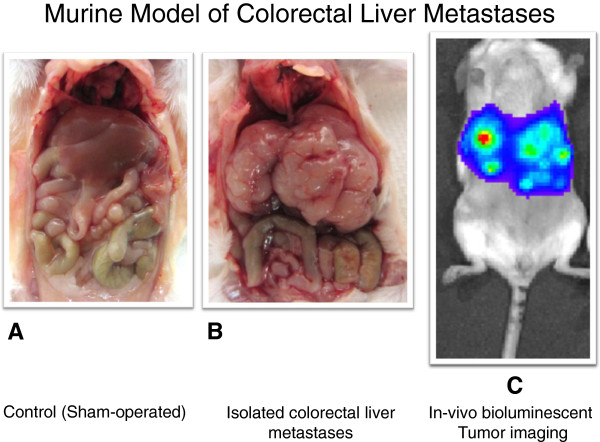
**Control mice underwent saline injection followed by splenectomy (A).** Isolated colorectal liver metastases formed in immunocompetent mice after intrasplenic injection of a syngeneic colorectal carcinoma line (**B**). Isolated colorectal liver metastases were confirmed by bioluminescence imaging prior to sacrifice (**C**).

**Figure 2:**
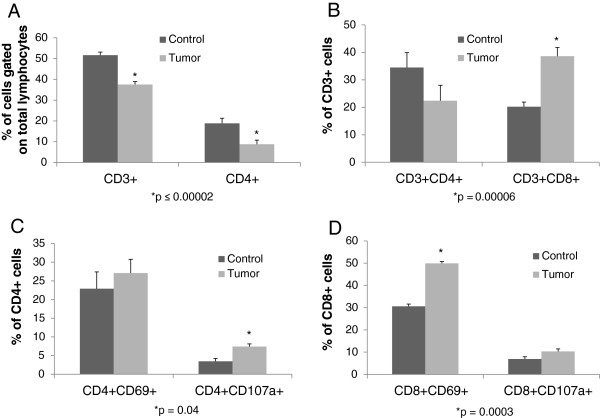
**Phenotype and function of T-cell subsets in colorectal liver metastases: Intrahepatic lymphocytes and tumor infiltrating lymphocytes were isolated.** Lymphocyte populations were decreased in the metastatic deposits compared to lymphocytes isolated from livers harvested from control animals, though the percentage of CD3 + CD8+ T-cells was increased, and overall reflected an activated phenotype. **A**. CD3+ cells as a percentage of total lymphocytes in metastatic tumor tissue was lower compared to the number of CD3+ T cells in corresponding control liver (mean ± SEM : 37.5% ± 1.4 vs. 51.6 % ± 1.5, p = 0.00001, n = 11 per group). CD4+ cells as a percentage of total lymphocytes in metastatic tumor tissue was lower compared to the number of CD4+ T cells in corresponding control liver (8.7% ± 1.9 vs. 18.8% ± 2.5, p = 0.00002, n = 11 per group). **B**. CD3 + CD4+ populations trended to be less in tumor deposits than normal liver (22.4% ± 5.6 vs. 34.5% ± 5.4, p = 0.14, n = 11 per group). CD3 + CD8+ cells were significantly increased in the CRLM (38.6% ± 3.2 vs. 20.2% ± 1.7, p = 0.00006, n = 11 per group). **C**,**D**. CD4 + CD107a + and CD8 + CD69+ populations were increased in CRLM (7.4% ± 0.7 vs. 3.5% ± 0.8, p = 0.04 ; 50% ± 0.9 vs. 30.6% ± 0.5 p = 0.0003, respectively, n = 3 per group) . There was no difference in CD4 + CD69+ cells between the groups and a trend towards increased CD8 + CD107a + expression on TIL compared to non-TIL (27.1% ± 3.6 vs. 22.9% ± 4.5, p = NS ; 10.3% ± 1.2 vs. 6.9% ± 0.6, p = 0.08 , respectively, n = 3 per group). CRLM = colorectal liver cancer metastases. TIL = tumor infiltrating lymphocytes.

Mice were sacrificed two weeks post operation after tumor burden and isolated colorectal liver metastases were confirmed by bioluminescence imaging (Figure 1). The number of CD3+ cells as a percentage of total lymphocytes in metastatic tumor tissue was lower compared to the number of CD3+ T cells in corresponding control liver (mean ± SEM : 37.5% ± 1.4 vs. 51.6 % ± 1.5, p = 0.00001). Similarly, CD4+ tumor infiltrating lymphocytes (TIL) as a percentage of total lymphocytes were decreased compared to CD4+ T cells in normal liver (8.7% ± 1.9 vs. 18.8% ± 2.5, p = 0.00002)(Figure 2A). There was no difference in total CD8+ cell populations between the tumor and control groups (11.7% ± 0.5 vs. 15.2% ± 1.3, p = 0.17). When CD4+ and CD8+ cell populations were determined as a percentage of CD3+ cells, CD3 + CD4+ populations again trended to be less in tumor deposits than normal liver (22.4% ± 5.6 vs. 34.5% ± 5.4, p = 0.14), but CD3 + CD8+ cells were significantly increased (38.6% ± 3.2 vs. 20.2% ± 1.7, p = 0.00006) (Figure 2B). There was no difference in CD4 + CD25 + Foxp3+ T regulatory cell populations between the groups, and their expression was rare (data not shown). Therefore, in CRLM, the percentage of CD3+ lymphocytes were decreased, CD3 + CD4+ cells trended to decrease, and CD3 + CD8+ TIL were increased compared to non-TIL from control livers.

To determine T cell activation phenotype, lymphocytes from both control liver and metastatic liver tumor tissue were evaluated for cell surface expression of the T cell activation markers CD69 and CD107a. CD4 + CD107a + and CD8 + CD69+ populations were increased in CRLM compared to populations in non-tumor liver tissue (7.4% ± 0.7 vs. 3.5% ± 0.8, p = 0.04 ; 50% ± 0.9 vs. 30.6% ± 0.5 p = 0.0003 , respectively) (Figure 2C). There was no difference in CD4 + CD69+ cells between the groups and a trend towards increased CD8 + CD107a + expression on TIL compared to non-TIL (27.1% ± 3.6 vs. 22.9% ± 4.5, p = 0.NS ; 10.3% ± 1.2 vs. 6.9% ± 0.6, p = 0.08 , respectively, Figure 2D). Therefore, TIL demonstrated a higher expression of T-cell surface markers indicative of an activated phenotype.

### Cytokine profiles within colorectal cancer liver metastases

**Figure 3:**
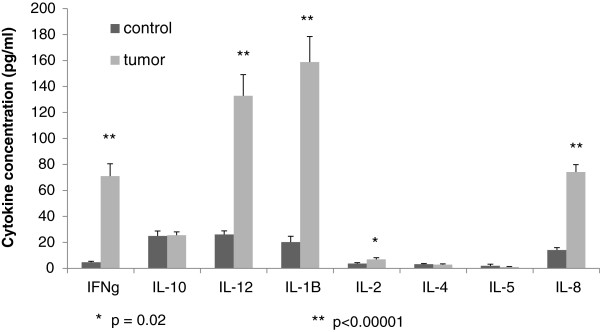
Cytokines in the tumor microenvironment of colorectal cancer liver metastases: Th1 cytokine responses dominated the immune milieu in metastatic tumors (n = 7) compared to control liver (n = 8).

To gain further insight on the immune microenvironment within colorectal cancer liver metastases, cytokine profiles were evaluated in both control liver and CRLM. Total protein was extracted from normal liver parenchyma and CRLM and normalized. Liver tumor tissue contained increased levels of the T-helper 1 (Th1) cytokines IFN gamma (p = 0.000008), IL-12 (p = 0.00002), IL-1b (p = 0.00001), and IL-8 (p = 0.0000002) compared to control liver tissue (Figure 3). There was no difference in T-helper 2 (Th2) cytokines (IL-4, IL-5, and IL-10) between the groups. The cytokine expression profile in the tumor microenvironment was indicative of Th1 cell-mediated immunity.

### LIGHT expression in colorectal cancer liver metastases

**Figure 4:**
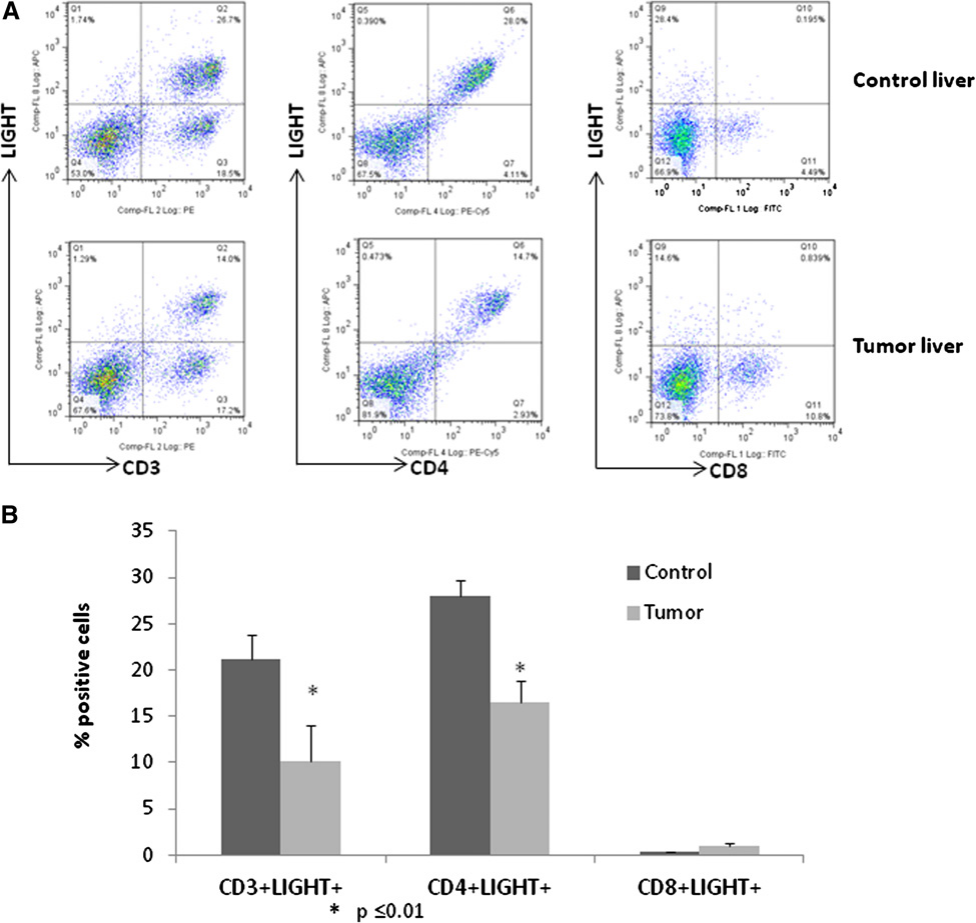
**LIGHT expression is decreased in CD3+ and CD4+ intratumoral lymphocytes. A**. Representative flow cytometry histogram of LIGHT expression in different T cell subsets. **B**. Summary of flow cytometry analysis of T cells from tumor tissues reveals decreased LIGHT expression on CD3+ and CD4+ lymphocytes. Twenty-one percent of control liver CD3+ cells were LIGHT + compared to 10.1% in CRLM (p = 0.017, n=7 per group). Similarly, 29% of control liver CD4+ cells were LIGHT + compared to 16.5% in CRLM (p = 0.007, n=3 per group).

We have previously identified intratumoral LIGHT gene expression as associated with survival after resection of CRLM, therefore, we sought to quantify and identify sources of LIGHT expression in healthy liver and in CRLM. When controlled for total lymphocyte cell populations, CD3 + T cells from CRLM had decreased LIGHT expression compared to CD3+ cells from control liver tissue (10.1% ± 4.0 vs. 21.1% ± 2.7, p = 0.017) (Figure 4). Similarly, expression of LIGHT was decreased in CD4+ T-cells from CRLM (16.5% ± 2.4 vs. 29% ± 1.7 vs., p = 0.007). LIGHT was expressed at very low levels by both intrahepatic and intratumoral CD8 + T-cells. In addition, LIGHT expression was decreased in both CD3+ (41% vs. 31%) and CD4+ (41% vs. 32%) T-cells and minimally expressed in CD8+ cells (<1%) from peripheral blood mononuclear cells in tumor bearing mice compared to controls.

### Immunofluorescence histochemistry analysis of LIGHT expression and lymphocytes in CRLM

**Figure 5:**
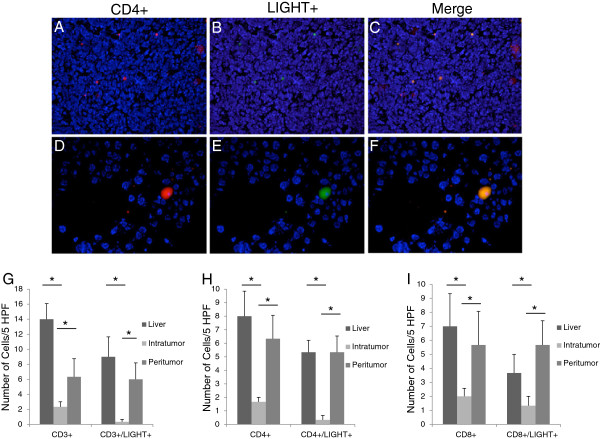
**Concurrent immunofluorescence staining of lymphocytes and LIGHT: Representative images of CD4+ staining (A,D red), LIGHT positive staining (B,E green) and co-expression (C,F yellow).** DAPI was utilized as a nuclear counterstain (blue). Upper panels at low power identify an area of CD4+ TIL infiltration in a resected colorectal liver metastasis (200x). Lower panels demonstrate a single high power field in a peritumoral specimen (400x). Intratumoral CD3+ (14 ± 2.08 vs. 2.33 ± .67, p = .00006), CD4+ (8 ± 1.86 vs. 1.67 ± 0.33, p = .0009) and CD8+ (7 ± 2.33 vs. 2.0 ± 0.58, p = .029) lymphocytes were decreased in CRLM compared to lymphocytes from corresponding and equal areas of healthy control liver. Total CD3 + LIGHT + (0.33 ± 0.33 vs. 9 ± 2.65, p = .00006), CD4 + LIGHT + (0. 33 ± 0.33 vs. 5.33 ± .88, p = .0009) and CD8 + LIGHT + (1.33 ± .67 vs. 3.67 ± 1.33, p = .029) cells were decreased in tumor bearing liver compared to control. LIGHT-expressing CD3+ (6.33 ± 2.40 vs. 0.33 ± .33, p = .00006), CD4+ (5.33 ± 1.20 vs. 0.33 ± .33, p = .0009), and CD8+ (5.67 ± 2.40 vs. 1.67 ± 0.33, p = .029) lymphocytes were significantly higher in the peritumor region compared to the intratumor region (panels **G**-**I**)(n = 6).

To define the areas in the tumor responsible for both T-cell infiltrate and LIGHT expression, immunofluorescence microscopy was performed. Total numbers of CD3+ (14 ± 2.08 vs. 2.33 ± .67, p = .00006), CD4+ (8 ± 1.86 vs. 1.67 ± 0.33, p = .0009) and CD8+ (7 ± 2.33 vs. 2.0 ± 0.58, p = .029) lymphocytes were decreased in CRLM compared to lymphocytes from corresponding and equal areas of healthy control liver (Figure 5). Also, concordant with FACS analysis, CRLM contained a decreased number of LIGHT expressing lymphocytes compared to non-tumor liver tissue. Total CD3 + LIGHT + cells were decreased in tumor bearing liver compared to control (0.33 ± 0.33 vs. 9 ± 2.65, p = .00006) (Figure 5). Similarly, CD4 + LIGHT + (0. 33 ± 0.33 vs. 5.33 ± .88, p = .0009) and CD8 + LIGHT + (1.33 ± .67 vs. 3.67 ± 1.33, p = .029) cells were decreased in tumor bearing liver compared to control (Figure 5). LIGHT expression was only present on lymphocytes and no LIGHT expression was identified on the CRLM or in the tumor stroma.

In regard to the architectural location of the T-cells in the tumor microenvironment, the number of LIGHT-expressing CD3+ (6.33 ± 2.40 vs. 0.33 ± .33, p = .000056), CD4+ (5.33 ± 1.20 vs. 0.33 ± .33, p = .0009), and CD8+ (5.67 ± 2.40 vs. 1.67 ± 0.33, p = .029) lymphocytes were significantly higher in the peritumor region compared to the intratumor region (Figure 5).

## Discussion

The presence of immune cells within primary colorectal tumors has been directly associated with improved clinical outcomes [19]. In particular, the presence of CD8+ TIL was associated with improved survival for patients with colorectal cancer, with 100% three-year survival of patients whose tumors contained a high density of intratumoral CD8 + TIL [20]. Similar to these observations made in primary colon tumors, the presence of increased CD4 and CD8 T cell infiltrates identified by immunohistochemistry in CRLM has also been associated with improved recurrence free and overall survival [8,9]. In another study, tumor infiltrating lymphocytes from resected human CRLM were found to respond against tumor antigens, in most patients, and flow cytometry of the TIL identified proportions of activated effector T-cells [21]. Furthermore, genetic ontology analysis of over 90 resected human CRLM identified T-cell activation as the most significant biologic function associated with recurrence free survival [10]. Based on these previous studies, increased T-cell infiltration into primary and metastatic colon cancer tumors is associated with improved survival and may be reflective of an anti-tumor and antigen-specific immune response. Therefore, immunostimulatory strategies that increase T cell infiltration into colorectal cancer liver metastases may be an ideal strategy for CRLM immunotherapy.

However, what is not clearly understood is how the immune microenvironment of CRLM differs from the rest of the liver, including determination of the phenotype, function, and location of TIL compared to intrahepatic lymphocytes. Furthermore, identification of the immunomodulatory cytokines present in the tumors, the type of cellular immunity generated by the tumor, and identification of potential targets that can be manipulated to increase the anti-tumor response remains to be elucidated. This gap in knowledge was investigated in order to characterize the CRLM immune microenvironment and identify novel immunotherapeutic strategies.

Immunotherapy has yielded successes in treating other human malignancies utilizing a variety of active and passive immunity strategies. Though in some cases tumor regression was obtained by adoptive transfer of TIL [22] or tumor specific antigen vaccines [23,24], tumor responses have also been achieved using immunostimulatory cytokines and antibodies that block immunosuppressive signals, e.g. CTLA4 [25-28]. For many cancers, therapy will likely transition toward a combination of immunotherapy and other targeted strategies to improve outcomes [29].

To determine the optimal immunotherapeutic strategy for CRLM, the tumor microenvironment must first be characterized. This has proven difficult secondary to the heterogeneity of metastatic patient tumors, the scarcity of TIL in colorectal cancer liver metastases, and inappropriate animal models. Though limited in that it may bypass certain biologic phenomena that occur during primary tumor progression, we have created a reliable model that addresses these issues and mimics many aspects of the human course of disease by utilizing a well established syngeneic murine model of CRLM [30,31].

Using this model, it was determined that, as in human CRLM, there were low levels of TIL overall and decreased levels of CD3 and CD4+ T-cells compared to non-tumor liver parenchyma. CRLM CD4+ subsets were decreased both in total and when calculated as a percentage of CD3+ cells, corresponding with decreased expression of CD3+ and CD4+ cells in CRLM on microscopy of independent tumor sections. Interestingly, numbers of CRLM CD8+ TIL were rare and not significantly different compared to non-tumor bearing liver populations when calculated as a percentage of total lymphocytes. However, when calculated as a percentage of CD3+ cells, a more accurate reflection of the percent of CD8+ cells (that also removed non-T-cell populations from the analysis), CD8+ cells were found to be significantly increased in the tumors. This would indicate infiltration of cytotoxic T-cells into the tumor microenvironment driven by tumor specific factors. Although it was demonstrated that the levels of CD4+ lymphocytes were decreased within CRLM compared to healthy liver, the CD4+ TIL were activated (increased CD4 + CD107a) compared to normal liver CD4+ cells, and these CD4+ TIL were perhaps driving the increased expression and activation of the CD8+ TIL. This is supported by cytokine analysis of the tumor microenvironment that reflected a Th1 cell-mediated immune response within the tumor with increased levels of IFN gamma, IL-12, IL-1, and IL-8 in the metastatic deposits. Therefore, it appears that there is an antigen driven, tumor specific cell-mediated immune response in CRLM, though brisk tumor infiltration was not present and the response was insufficient to eliminate the tumors.

When evaluated by immunofluorescence, we found that the majority of the CD8+ cells were found in the peritumoral tissues. Phenotypic analysis of these TIL, however, found them to be significantly activated over CD8+ cells not in the tumor microenvironment or in non-tumor bearing livers. Therefore, it is possible that the tumor stroma and a number of other immunoediting mechanisms employed by the tumor limit the ability of CD8+ cells to infiltrate into the tumor nests [20,32]. Therefore, mechanisms that either increase TIL numbers and their activation status, or allow greater intratumoral infiltration may be a specific viable therapeutic strategy.

LIGHT is an immunostimulatory cytokine that has been shown to increase the number of TIL and their activation status, and to induce tumor regression via upregulation of the antitumor immune response [13]. Though this has not been demonstrated in CRLM, our gene ontology analysis of resected CRLM identified an association between increased LIGHT expression and survival [10]. Therefore, we were interested in characterizing LIGHT expression in the tumor microenvironment as a potential therapeutic target. Although FACS analysis of cell surface markers demonstrated that the TIL were significantly activated compared to intrahepatic lymphocytes, the TIL expressed significantly less LIGHT. It is important to note that while there was a pattern of increased LIGHT + staining in control liver tissue compared to *intra*tumoral tissue, the same decrease was not present when comparing control liver tissue to *peri*tumor liver tissue (liver tissue immediately adjacent to the metastases). Although the number of total T-cells was lower in peritumor liver tissue compared to control liver, the levels of LIGHT + T cells were similar, if not comparable. Therefore, there appears to be a barrier to LIGHT expression within intratumoral TIL and this may be suppressing a tumor specific immune response or may reflect a tumor immune-escape mechanism [33,34].

In summary, characterization of the tumor microenvironment of CRLM revealed that although a limited number of activated T-cells are infiltrating the tumor and initiating an immune response, the number of LIGHT + T cells infiltrating the tumor are very low. Considering LIGHT has previously been shown to cause proliferation and activation of T cells, it is possible that an increase in LIGHT + T cells within the tumor could amplify the existing immune response.

Limitations of this analysis include the use of an animal model of disease rather than human tissues. However, the heterogeneity of patient factors and variable growth and tumor architecture of patient-derived tumors can often limit the analysis, and this syngeneic immunocompetent model allows consistent and reproducible tumors with less heterogeneity. Reproducibility of the results in different syngeneic cell lines on alternate murine backgrounds is needed. Furthermore, use of sham –operated murine liver tissue to compare to tumor bearing liver limits analysis to intrahepatic lymphocytes versus TIL. This was felt to be a more appropriate comparison than to use apparently uninvolved or healthy appearing liver from the experimental mouse liver, as micrometastatic disease may have been present. Ideally, TIL from CRLM would be compared to TIL from benign lesions or to CRLM treated with immunomodulatory agents. It is critical, however, to define the tumor microenvironment at baseline compared to normal liver tissue, in order to identify targets for future studies. What is evident from recent data and our observations in the fixed tissues was that critical and unique immunological interactions may be occurring in the peritumoral milieu and stroma. For this reason, we critically evaluated the peritumoral area on fixed tissue slides since the domain could be identified and defined with microscopy (Figure 5). It was not possible to accurately define or isolate sufficient numbers of lymphocytes from the small rims of peritumoral tissue around very small murine liver metastases, from a technical perspective, therefore, FACS analysis was not performed separately on peritumoral tissues. In addition, it is possible that the size of the metastases influenced the amount of TIL in the lesions. There was variation in the size of the lesions within the same liver, as one would also expect in the human course, however, the variations were relatively similar between animals. TIL from the same animal were pooled and compared to TIL from control animals, therefore, this should have decreased the bias of the size of any individual metastatic lesion. Furthermore, it would also have been interesting to analyze additional T-cell subsets, however, phenotypic and functional determination by flow cytometry is limited in scope by the number of TIL that are in the tumors. We did further evaluate CD3 + CD4-CD8- T-cells utilizing the murine pan-NK cell marker CD49B and speculate that many of the uncommon T-cells are natural killer and γδT cells, acknowledging that the liver is a reservoir for double negative T-cells [35]. Further analysis of LIGHT levels in these cells and in γδT cells are planned. Despite these limitations, the findings of this study lay the foundation for evaluation of future immunotherapies and validation studies with the goal of inciting clinical regressions of established CRLM.

## Conclusions

The success of immunotherapy in other diseases, and the critical finding that colorectal cancer patients with increased TIL live longer, has identified immunotherapy as a potential optimal strategy for patients with CRLM. Our current gap in knowledge is a lack of understanding of the immune microenvironment of CRLM, and in identifying potential immunostimulatory interventions.

We have herein characterized the phenotype and function of TIL in the tumor microenvironment using an immunocompetent syngeneic animal model of CRLM, and have defined the expression of LIGHT, a potential immunotherapeutic agent for this disease. Based on the cytokine milieu, there appears to be Th1 cell-mediated immunity within CRLM that is supporting a CD8+ cytotoxic T-lymphocyte based tumor specific immune response. The number of CD3 + CD4+ TIL is limited, however the percent of CD3 + CD8+ TIL is increased, and these cells have an activated phenotype. Importantly, the majority of CD8+ cells in the tumor microenvironment were peritumoral, and LIGHT was barely detectable on most CD8+ TIL, which supports a mechanism of immunosuppression on the TIL that are migrating to and infiltrating into the metastatic tumors. Techniques to decrease suppressive influences or augment the cytotoxic T-cell response are needed and may be possible through mechanisms that can increase intratumoral LIGHT + TIL. Future studies are focused on evaluating changes in the tumor microenvironment through increased activation and stimulation of TIL, including with LIGHT.

## Competing interests

The authors declare that they have no competing interests.

## Authors’ contributions

JQ carried out phenotypic and functional studies of harvested T-cell subsets, cytokine profiling of CRLM, LIGHT flow cytometry, and co-drafted the manuscript. VU performed immunofluorescence histochemistry analysis of LIGHT expression and co-drafted the manuscript. BP participated in the coordination of the study and editing of the manuscript. AVM conceived the study, designed the experiments, performed the animal surgeries, and drafted the manuscript. All authors read and approved the final manuscript.
